# CpG Methylation Changes G-Quadruplex Structures Derived from Gene Promoters and Interaction with VEGF and SP1

**DOI:** 10.3390/molecules23040944

**Published:** 2018-04-18

**Authors:** Kaori Tsukakoshi, Shiori Saito, Wataru Yoshida, Shinichi Goto, Kazunori Ikebukuro

**Affiliations:** 1 Department of Biotechnology and Life Science, Tokyo University of Agriculture and Technology, 2-24-16 Naka-cho, Koganei, Tokyo 184-8588, Japan; k-tsuka@cc.tuat.ac.jp (K.T.); s166099v@st.go.tuat.ac.jp (S.S); gotoshinichi2000@gmail.com (S.G.); 2 School of Bioscience and Biotechnology, Tokyo University of Technology, 1404-1 Katakuramachi, Hachioji, Tokyo 192-0982, Japan; yoshidawtr@stf.teu.ac.jp

**Keywords:** G-quadruplex, CpG methylation, vascular endothelial growth factor, SP1, protein-DNA binding

## Abstract

G-quadruplex (G4) is a DNA/RNA conformation that consists of two or more G-tetrads resulting from four-guanine bases connected by Hoogsteen-type hydrogen bonds, which is often found in the telomeres of chromatin, as well as in the promoter regions of genes. The function of G4 in the genomic DNA is being elucidated and some G4-protein interactions have been reported; these are believed to play a role in vital cellular functions. In this study, we focused on CpG methylation, a well-known epigenetic modification of the genomic DNA, especially found in the promoter regions. Although many G4-forming sequences within the genomic DNA harbor CpG sites, the relationship between CpG methylation and the binding properties of associated proteins remains unclear. We demonstrated that the binding ability of vascular endothelial growth factor (*VEGF*) G4 DNA to VEGF165 protein was significantly decreased by CpG methylation. We identified the binding activity of G4 DNA oligonucleotides derived from gene promoter regions to SP1, a transcription factor that interacts with a G4-forming DNA and is also altered by CpG methylation. The effect of methylation on binding affinity was accompanied by changes in G4 structure and/or topology. Therefore, this study suggested that CpG methylation might be involved in protein binding to G4-forming DNA segments for purposes of transcriptional regulation.

## 1. Introduction

G-quadruplex (G4) is a non-canonical nucleic acid structure formed in G-rich sequences [1]. G4 is formed by stacking of G-tetrads to form a planar array of four guanine bases connected by Hoogsteen bonding. It is well-known that the thermostability of G4 structures depends on the concentration and type of monovalent cations [2]. Telomeric DNA folds into a G4 structure to maintain telomere length [3]. The topology of human telomeric DNA depends on the type of cations: G4 folds into a basket-type mixed antiparallel/parallel -stranded G4 structure or hybrid-type mixed antiparallel/parallel-stranded G4 structure in the presence of Na^+^ or K^+^, respectively [4,5]. These results suggest that the topology and stability are related to the biological function of G4. G4-forming DNAs have also been identified in several promoter regions [6,7,8] and are involved in transcriptionally activating [9] or silencing gene expression [10,11,12]. Moreover, genome-wide G4-forming DNA identification analysis revealed that G4-forming DNAs are enriched in gene regulatory elements, including gene promoters and CpG islands in genomic DNA [13,14,15,16,17,18,19,20].

Several G4-binding proteins have been identified such as CNBP and nucleolin [21]. We previously reported that the vascular endothelial growth factor 165 (VEGF165) protein binds to G4 DNA formed in the *VEGF* promoter [22] and G4 RNA formed in *VEGF* pre-mRNA [23]. Moreover, it has been reported that specificity protein 1 (SP1), a C_2_H_2_-type zinc finger transcription factor bound to the *c-KIT* G4 structure with an affinity similar to that of the duplex structure [24]. The consensus sequence of SP1 is [5′-(G/T)GGGCGG(G/A)(G/A)(G/T)-3′] [25], and SP1 is ubiquitously expressed to not only maintain basal transcription of housekeeping genes, but also regulate tissue-specific gene expression [26]. In several cancers, SP1 is often overexpressed and the target genes are mainly involved in oncogenesis [27]. Genome-wide analysis revealed that SP1 binding sites were enriched in the promoter and CpG island [28]. In silico analysis revealed a correlation between G4-forming sequences upstream of genes and the occurrence of the SP1-binding element [29,30]. These results suggest that G4 is involved in controlling gene expression by SP1.

DNA methylation is an epigenetic modification recognized by methylated CpG binding proteins to recruit the enzymatic machinery needed to establish silent chromatin [31,32,33]. DNA methylation affects not only the thermodynamic properties of DNA duplex structures [34,35] but also G4 structures. For example, the thermostability of quadruplex structures formed by d(CGCG_3_GCG) oligonucleotides [36], *FMR1* repeats [37], *C9orf72* repeats [38], and *BCL-2* G4 [39] is increased by DNA methylation, whereas the thermostability of *MEST* G4 is decreased by DNA methylation [40]. We previously reported that the initial elongation efficiency of PCR decreased when template DNA containing a *VEGF* G4-forming sequence was methylated, suggesting that the thermostability of *VEGF* G4 was increased by CpG methylation [41]. These results suggest that the thermostability and topology of G4 structures are affected by CpG methylation, resulting in altered binding ability of G4-binding proteins to G4 structures. In this study, we investigated the binding ability of VEGF165 to methylated *VEGF* G4 DNA [42,43] and the binding ability of SP1 to methylated *BCL-2*, *VEGF*, *c-KIT*, *HRAS1*, and *HRAS2* G4 structures [44,45,46]. 

## 2. Results

### 2.1. Effect of CpG Methylation of VEGF G4 DNA on Binding Ability for VEGF165

In this study, unmethylated and methylated 20-mer oligonucleotides of *VEGF* DNA containing three CpG sites were analyzed (Table 1). We previously reported that VEGF165 protein bound to the *VEGF* G4 DNA in vitro [22]. Binding domain analysis revealed that *VEGF* G4 bound to the heparin-binding domain of VEGF165. The G4 structure has twice as much negative charge density as the double-stranded DNA structure [47] and the heparin-binding motif contains several basic amino acid residues that interact with the negatively charged sulfo groups on heparin. These results suggest that G4 structures preferentially bind to the heparin-binding domain via electrostatic interactions.

**Figure 1:**
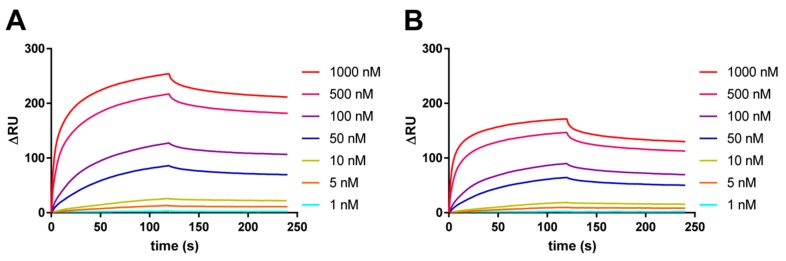
Binding analysis of VEGF165 to *VEGF* G4 DNA by SPR. Representative SPR binding signal of unmethylated (**A**) or methylated *VEGF* G4 DNA (**B**) to VEGF165 immobilized on a CM5 chip.

To investigate whether the interaction is affected by CpG methylation of the G4 structure, surface plasmon resonance (SPR) binding analysis was performed and binding constant (*K*_D_) was elucidated. The VEGF165 protein was immobilized on a CM5 sensor chip and then unmethylated or methylated *VEGF* G4 DNA was injected onto the VEGF165-immobilized chip. As previously reported, binding activity of unmethylated *VEGF* G4 to VEGF165 protein was confirmed, with a *K*_D_ of 13 ± 0.43 nM (Figure 1). In contrast, the *K*_D_ of the methylated *VEGF* G4 to VEGF165 protein increased to 25 ± 5.7 nM, indicating that binding activity was decreased by DNA methylation.

Methylated CpG sites are underlined. For the plate assay, G4 DNAs were biotinylated at the 5′ end for immobilization to streptavidin-coated wells. For Native-PAGE, G4 DNAs were modified with TAMRA at the 3′ end for detection of electrophoretogram.

### 2.2. Effect of CpG Methylation of Promoter-Derived G4-Forming DNA on DNA-Binding Ability of GST-SP1

**Figure 2:**
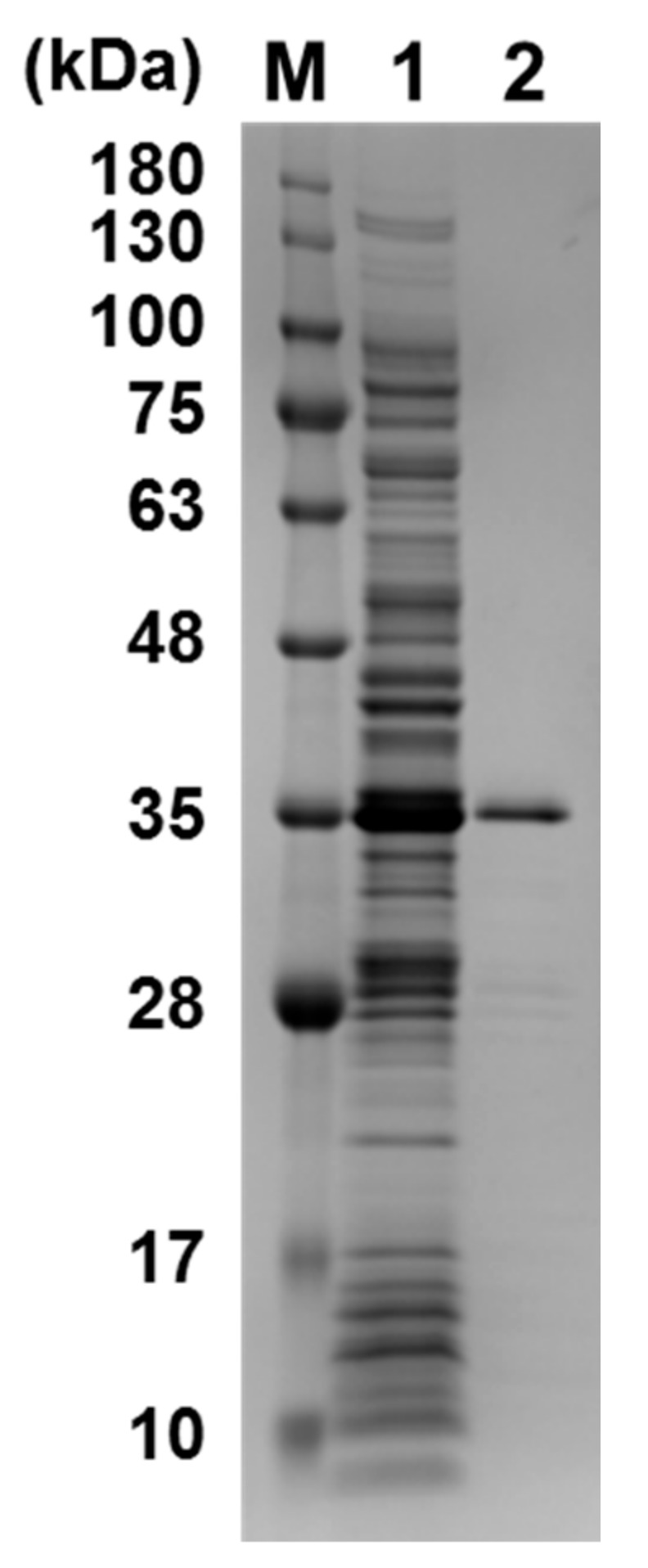
SDS-PAGE of purified GST-SP1 (37.5 kDa). M: protein marker; 1: crude fraction; 2: purified GST-SP1.

**Figure 3:**
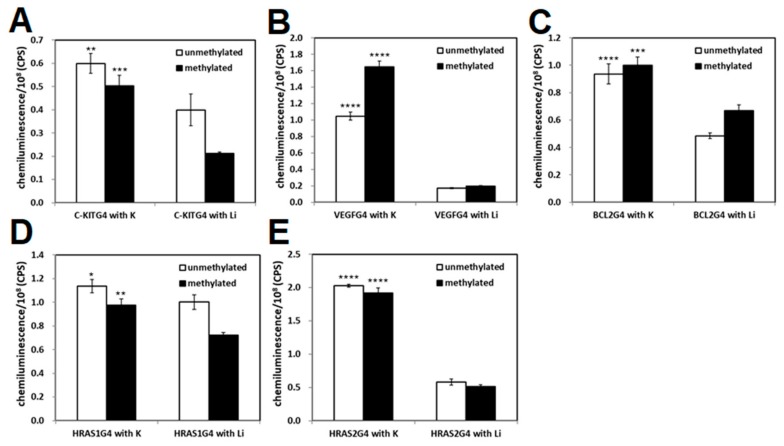
Binding ability of SP1 to *c-KIT* (**A**); *VEGF* (**B**); *BCL-2* (**C**); *HRAS1* (**D**); and *HRAS2* (**E**) G4 DNAs in the presence of K^+^ or Li^+^. Binding of GST-SP1 to G4 DNAs immobilized on the well was detected with an HRP-conjugated anti-GST antibody. As a negative control, a well without immobilized DNA was utilized. Bar plots mean values ± SD (N = 3). Statistical analyses were performed using two-way ANOVA with Tukey’s multiple comparisons post-hoc tests. The significance of differences of observed values from the Li^+^-treated group are indicated as follows: * *p* < 0.05; ** *p* < 0.001; *** *p* < 0.0005; **** *p* < 0.0001.

We tested the binding ability of methylated and ummethylated 22-mer *c-KIT*, 20-mer *VEGF*, 27-mer *BCL-2*, 27-mer *HRAS1,* and 24-mer *HRAS2* G4-forming DNAs, which contain 3, 4, 4, and 5 CpG sites, respectively, to the transcription factor GST-SP1. In the *c-KIT* promoter region, three G4-forming sequences were identified and the middle G4-forming sequence contains an SP1 binding motif; therefore, the middle *c-KIT* G4-forming DNA was used. SP1 protein was expressed as a GST fusion protein in *E. coli* as previously reported [48]. The purified GST-SP1 was confirmed by SDS-PAGE (Figure 2). Enzyme-linked oligonucleotide assay (ELONA) revealed that a stronger chemiluminescent signal from HRP was observed to come from the wells with all G4 DNAs in comparison with the negative control wells not harboring G4 DNAs (Figure 3A–E). It was also confirmed that no binding occurred between the GST tag and G4 DNA (data not shown). To investigate the effect of G4 folding on the binding ability of SP1, the potassium ions in the folding buffer were substituted with lithium ions, which do not promote the formation of G4. The binding of SP1 to all G4 DNAs in the presence of Li^+^ was significantly decreased, indicating that SP1 recognized G4 folding of single-stranded *c-KIT*, *BCL-2*, *VEGF*, *HRAS1*, and *HRAS2* DNAs regardless of the presence of methylated CpG sites (Figure 3A–E). In the presence of K^+^, methylated *VEGF* G4 DNA exhibited a 1.5 times stronger chemiluminescent signal from SP1 binding activity than unmethylated *VEGF* G4 DNA did, whereas the binding signal in the presence of Li^+^ was not increased by DNA methylation (Figure 3B). Based on these results, we can conclude that SP1 interacted with the five G4-forming DNAs derived from the promoter regions of their genes—specifically *c-KIT*, *BCL-2*, *VEGF*, *HRAS1*, and *HRAS2*. Furthermore, it can be concluded that these binding activities between these G4-forming DNAs and SP1 could be affected by CpG methylation of them.

An SPR experiment was performed to elucidate the *K*_D_ of G4-forming oligonucleotides to SP1 (Figure S1). The binding ability (i.e., *K*_D_) of all the G4-forming DNAs to GST-SP1 was confirmed (Table 2). We compared the *K*_D_ of G4 DNA containing methylation at CpG sites to that of ummethylated G4 DNA. Lower *K*_D_ values were obtained for *c-KIT* and *VEGF* G4 DNA that had undergone CpG methylation compared with their unmethylated forms. The CpG methylation of *BCL-2*, *HRAS1*, and *HRAS2* G4 DNAs had the opposite effect, and resulted in higher *K*_D_ values of those G4-forming DNAs when methylated than unmethylated. The binding ability of SP1 to G4-forming DNA increased or decreased by CpG methylation depending on the gene investigated, implying that CpG methylation may function as both a positive or negative regulator of protein-G4 structure binding. This result was in agreement with experimental observations from our study of *VEGF* G4 DNA binding to VEGF165 protein (Figure 1).

### 2.3. Analysis of G4 Folding in the Presence or Absence of CpG Methylation

**Figure 4:**
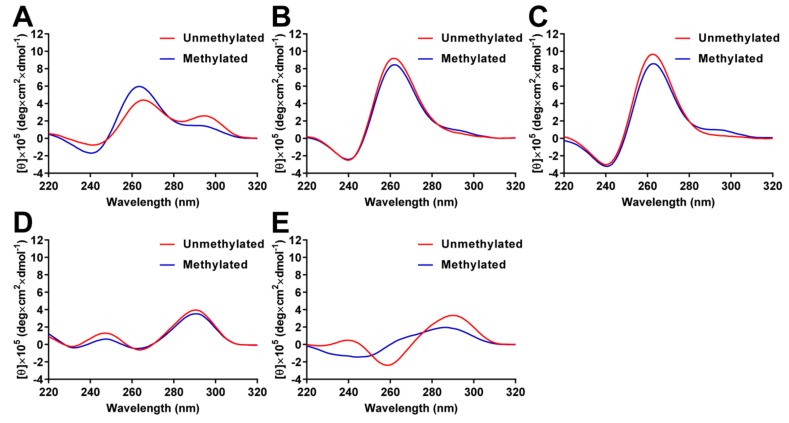
CD spectra of unmethylated and methylated *BCL-2* (**A**); *VEGF* (**B**); *HRAS2* (**C**); *c-KIT* (**D**); and *HRAS1* G4 DNAs (**E**) in 10 mM Tris-HCl, 100 mM KCl, pH 7.4 at 25 °C.

**Figure 5:**
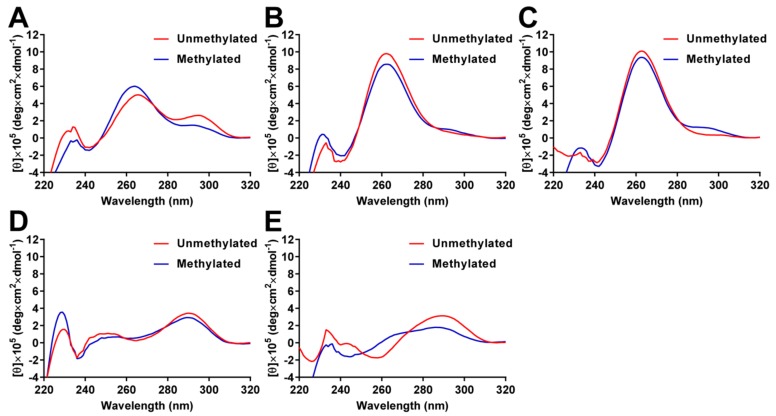
CD spectra of the unmethylated and methylated *BCL-2* (**A**); *VEGF* (**B**); *HRAS2* (**C**); *c-KIT* (**D**); and *HRAS1* G4 DNAs (**E**) in the presence of the GST-SP1 in 10 mM Tris-HCl, 100 mM KCl, pH 7.4 at 25 °C.

To analyze the effects of CpG methylation on the G4 structures, the circular dichroism (CD) spectra were measured in 10 mM Tris-HCl (pH 7.4) containing 100 mM KCl. CD spectra of unmethylated *BCL-2* G4 showed two positive peaks at 265 and 295 nm; the peak at 295 nm was decreased by CpG methylation (Figure 4A). These results indicate that unmethylated *BCL-2* G4 folds into a mixed-type G4 structure, while methylated *BCL-2* G4 mainly folds into a parallel G4 structure, as previously reported [39]. It has been reported that *VEGF* and *HRAS2* G4 DNAs fold into parallel G4 structures and *c-KIT* and *HRAS1* G4 DNAs fold into anti-parallel G4 structures in 100 mM KCl. The topology of unmethylated *VEGF* and *HRAS2* G4 DNAs were confirmed by CD spectral analysis, as *VEGF* and *HRAS2* G4 DNA showed a positive peak at approximately 262 nm, which is typical for a parallel G4 structure (Figure 4B,C). In contrast, the positive peaks decreased and a shoulder between 280 and 300 nm was detected by CpG methylation, indicating that methylated G4 DNAs were present. Unmethylated *c-KIT* and *HRAS1* G4 DNAs showed a positive peak at approximately 290 nm, which indicates a typical fold into a parallel G4 structure, but antiparallel topology spectra for an anti-parallel G4 structure (Figure 4D,E). By methylating *c-KIT* G4 DNA, the positive peak at approximately 290 nm decreased, indicating that methylated *c-KIT* G4 DNA folds into an anti-parallel G4 structure that differs from the unmethylated structure. For *HRAS1* G4 DNA, the positive peak at 290 nm was shifted to 286 nm and the negative peak at 259 nm and positive peak at 239 nm were not detected after DNA methylation, suggesting that methylated *HRAS1* G4 does not fold into a G4 structure. We measured the CD spectra of G4 DNAs in the presence of GST-SP1 to investigate whether SP1 recognizes the G4 structures or single-stranded DNA structures. CD spectra of both unmethylated and methylated G4 DNAs were not affected by addition of GST-SP1 (Figure 5), indicating that G4 DNAs folded into the G4 structure in the presence of GST-SP1 and SP1 recognized the G4 structures of *c-KIT*, *BCL-2*, *VEGF*, *HRAS1*, and *HRAS2*. These results indicated that methylation of cytosine in G4 structures gave rise to different G4 topology, whereby the binding abilities of VEGF165 and SP1 to the G4 DNAs were altered by methylation.

**Figure 6:**
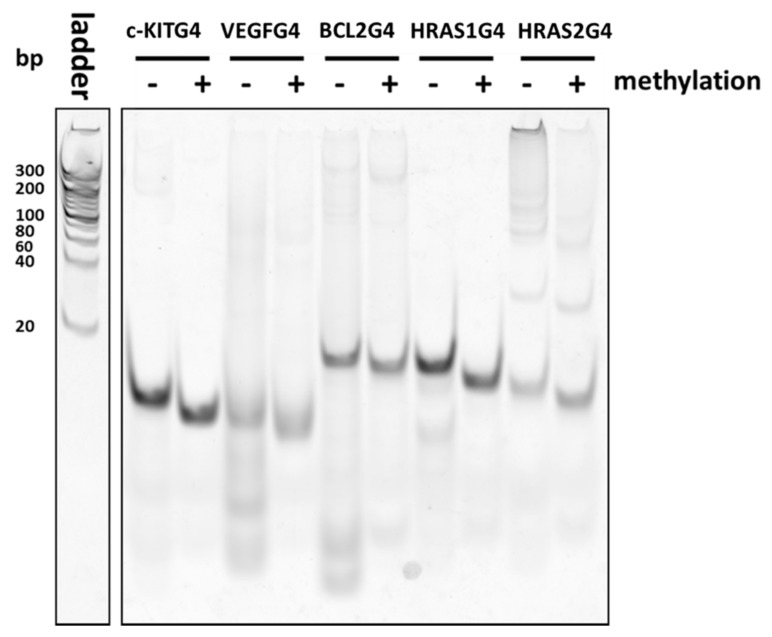
Native-PAGE of the unmethylated and methylated *c-KIT*, *BCL-2*, *VEGF*, *HRAS1*, and *HRAS2* G4 DNAs. Oligonucleotides fluorescently labelled with TAMRA were detected. Ladder: DNA marker.

Native-PAGE analysis showed that CpG methylation of DNA within G4 structures caused distinct mobility in the gel (Figure 6). This was especially apparent when methylated *VEGF*, *BCL-2*, and *HRAS2* G4 DNAs separated into multiple bands, which were distinct from those of the unmethylated DNAs. Thus, G4-associated DNAs can fold into many different types of G4 types, as observed among the G4 DNAs in this study. G4-forming sequences can fold into several types of structures with differing topology, multimer components, or structures folded by different guanine bases. Interestingly, even in the presence of the complementary strand of each G4-forming DNA, we observed multiple bands in Native-PAGE (Figure S2). Some SP1-binding sites that are generally thought to form dsDNA therefore seem to form different, distinct structures apart as ssDNA from those formed while in the dsDNA state.

## 3. Discussion

In this study, we demonstrated that CpG methylation in G4 DNA structures was involved in mediating the binding ability of VEGF165 and SP1 to G4 DNAs. The affinity of VEGF165 to *VEGF* G4 DNA was decreased by CpG methylation. The binding of GST-SP1 to *c-KIT*, *BCL-2*, *VEGF*, *HRAS1*, and *HRAS2* G4 DNAs were confirmed by ELONA and SPR to have *K*_D_ values of 10–36 nM, which are altered by the presence of methylated cytosine. CD spectra analysis indicated that the topology of different G4 showed different spectra, with some change to these by CpG methylation in all G4 DNAs. Additionally, the electrophoretogram showed that the CpG methylation altered the distribution of several states of G4. These results suggested that the effect of CpG methylation on binding activity of the proteins studied to G4 DNA led to changes in the mixed states of G4 structures. We have previously reported on DNA aptamers, which fold into some G4 structures different from typical G4 topology, were able to increase the affinity for target molecules (e.g., proteins) by changing G4 topology in such a way to make it more suitable for binding by them [49]. Thus, among the several different states of a given G4 DNA, there should be a structure that has high binding ability for GST-SP1. If the mixed states were altered by DNA methylation, the structure binding to GST-SP1 was increased/decreased by the cytosine methylation. This might result in the enhancement/inhibition of binding ability of G4 DNA against GST-SP1. Methylation at the C-5 position of cytosine increases the molecular polarizability of pyrimidine to enhance stacking interactions [36]. Moreover, hydrophobic methyl groups on cytosine affect the electrostatic potential of the major groove [50] and methylation may add CH-π interactions, which have important roles in the G4 structure [51]. It was suggested that the CH-π interactions by methylation can stabilize structure or topology of G4. These properties would affect not only the G4 structure, but also the protein binding activity.

It has been reported that the binding activity of SP1 to double-stranded DNA containing the consensus sequence was not affected by DNA methylation [52,53]. We determined SP1 recognized the G4 structures of *c-KIT*, *BCL-2*, *VEGF*, *HRAS1*, and *HRAS2* and its bindings may be caused by the change of mixed G4 states by DNA methylation, suggesting that G4 structure containing CpG methylation site is involved in transcriptional regulation. DNA methylation on promoters is generally recognized as an epigenetic modification for transcriptional repression, as CpG binding proteins bind to methylated regions to recruit the enzymatic machinery needed to establish silent chromatin. In contrast, DNA methylation profiling analyses have identified hypermethylated low-CpG content promoters with transcriptional activity [54,55], suggesting that the regions are in an open chromatin state. CpG methylation profiling analyses also indicated that 31% of experimentally verified SP1 binding sites were hypermethylated, suggesting that SP1 binding occurs independently of methylation [54]. These results suggest that G4 structures are formed in methylated promoter regions when the regions are in an open chromatin state and that transcriptional activity is regulated via SP1 binding to methylated G4 structures. We also expect that CpG methylation in *VEGF* G4 DNA could have some roles in cell function, which are controlled by its binding to the VEGF protein.

## 4. Materials and Methods

### 4.1. Expression and Purification of GST Fused SP1

*Escherichia coli* BL21 (DE3) were transformed with pGEX2T-SP1 [48]. GST-SP1 fusion protein was expressed using the Overnight Express™ autoinduction system at 20 °C for 30 h. The cell pellets were collected by centrifugation at 3000× *g* for 10 min and resuspended in cell lysis buffer (10 mM Na_2_PO_4_, 1.8 mM KH_2_PO_4_, 140 mM NaCl, 2.7 mM KCl, 90 µM ZnCl_2_, 5 mM DTT, 4 mM Pefabloc, 1% Triton X-100, pH 7.3). The sample was homogenized using a French press (Ohtake, Tokyo, Japan) and centrifuged at 8000× *g* for 30 min at 4 °C. The supernatant was filtered through a 0.2-µm nitrocellulose filter (Advantec, Dublin, CA, USA). The filtered sample was loaded onto two tandemly connected GSTrap HP 1 mL columns (GE Healthcare, Little Chalfont, UK) to purify GST-SP1. The column was washed with 10 mL of wash buffer (10 mM Na_2_PO_4_, 1.8 mM KH_2_PO_4_, 140 mM NaCl, 2.7 mM KCl, 90 µM ZnCl_2_, 5 mM DTT, pH 7.3). GST-SP1 was eluted using 8 mL of elution buffer (50 mM Tris-HCl, 10 mM reduced glutathione, 90 µM ZnCl_2_, 5 mM DTT, 4 mM Pefabloc, pH 8.0). The purified sample was analyzed on a 12% sodium dodecyl sulfate–polyacrylamide gel electrophoresis (SDS-PAGE) gradient gel (WAKO, Osaka Japan) and visualized with Coomassie Brilliant Blue (Kanto Chemical Co., Inc., Tokyo, Japan). The purified protein was dialyzed against TBS buffer (10 mM Tris-HCl, 150 mM NaCl, 5 mM KCl, 90 µM ZnCl_2_). Protein concentration was measured by using Bradford reagent (Sigma, St. Louis, MO, USA). 

### 4.2. Binding Analysis of VEGF165 to VEGF G4 DNAs by SPR

All oligonucleotides employed in this study (Table 1) were synthesized elsewhere (Eurofins Genomics K.K., Tokyo, Japan; TAKARA BIO INC., Shiga, Japan). Approximately 1500 RU of VEGF165 was immobilized on a sensor chip CM5 (GE Healthcare, Little Chalfont, UK) using an amine coupling procedure in 10 mM acetate buffer (pH 6.0). The oligonucleotides were diluted to 10 µM in TK buffer (10 mM Tris-HCl, 100 mM KCl, pH 7.4). These DNA samples were denatured at 95 °C for 10 min and then allowed to cool to 25 °C for 30 min. Various concentrations of oligonucleotides were injected onto the VEGF165-immobilized chip at a flow rate of 30 μL/min at 25 °C. The VEGF165-immobilized chip was regenerated by injection of a mixture of 1 M NaCl and 1 mM NaOH. Dissociation constant (*K*_d_) values were calculated by fitting the association and dissociation rates using BIA evaluation software (GE Healthcare). SPR binding analysis was performed four times to evaluate the *K*_d_ value (mean ± SD, N = 4).

### 4.3. Binding Analysis of SP1 to Methylated G4 DNAs by ELONA and SPR

The oligonucleotides were modified with biotin at the 5′ end. The biotinylated oligonucleotides (1 µM) were folded by heat treatment in TK buffer or Tris-LiCl buffer (10 mM Tris-HCl, 100 mM LiCl, pH 7.4) as described above. The oligonucleotides were diluted in rinse buffer (TBS (10 mM Tris-HCl, 150 mM NaCl, 5 mM KCl), 90 µM ZnCl_2_, pH 7.3) to 250 nM and then 100 µL of the diluted oligonucleotide samples were added to a streptavidin-coated 96 well plate (Nalge Nunc International, Rochester, NY, USA). After washing the wells with 100 µL of washing buffer (TBS, 0.05% Tween 20, 90 µM ZnCl_2_, pH 7.3), 100 µL of GST-SP1 (250 nM) was added and then incubated for 1 h at RT. After washing the wells with 100 µL of washing buffer four times followed by 100 µL of rinse buffer twice, 100 µL of 10,000-fold diluted horseradish peroxidase (HRP)-conjugated anti-GST antibody (Abcam, Cambridge, UK) was added. After 1 h incubation at RT, the wells were washed with 100 µL of the washing buffer six times followed by 100 µL of rinse buffer twice, and then 100 µL of SuperSignal ELISA Pico Chemiluminescent Substrate (Thermo Fisher Scientific, Waltham, MA, USA) was added. The chemiluminescence intensities were measured at RT with a microplate reader (Perkin Elmer, Waltham, MA, USA). 

For binding analysis of SP1 to double-stranded DNAs, the oligonucleotide that could form G4 was modified with biotin at the 5′ end, while the complementary strand of G4-forming oligonucleotide was not biotinylated. Both oligonucleotides were mixed and folded by heat treatment in TK buffer as described above. Final concentrations of both oligonucleotides were 1 µM. We confirmed the formation of double-stranded structures by electrophoresis (data not shown).

In SPR analysis, an anti-GST antibody was immobilized on a CM5 sensor chip, and then GST tag or GST-SP1 were captured on a reference cell or a flow cell, respectively. *VEGF*, *c-KIT*, *BCL-2*, *HRAS1*, and *HRAS2* G4 DNAs were injected onto the sensor chip. *K*_D_ of G4 DNAs against GST-SP1 was determined using a single-cycle kinetics approach. A dilution series of G4 DNAs was prepared at concentrations of 10, 20, 40, 80, and 160 nM.

### 4.4. Circular Dichroism Spectroscopy

The oligonucleotides (1 µM) were folded by heat treatment in TK buffer as described above. GST-SP1 (final concentration (f.c.) 0 or 1.5 µM) was added to the DNA samples (f.c. 1.5 µM) and incubated for 30 min at room temperature. Circular dichroism (CD) spectra was measured using a J-820 spectropolarimeter (JASCO, Tokyo, Japan) and a quartz cell of 10 mm optical path length (JASCO) at 25 °C. 

### 4.5. Native-PAGE Analysis

The G4 DNAs were modified with addition of a TAMRA label at the 3′ end. The folded oligonucleotides (f.c. 500 nM) in TK buffer were separated in a 20% polyacrylamide gel and visualized with fluorescence of TAMRA. To analyze dsDNA forms of the G4 DNA, the complementary strand of each G4-forming DNA was mixed in just before heat treatment.

## Figures and Tables

**Table 1:** DNA sequences used in this study.

Name	Sequence (5′–3′)
*VEGF* G4	GGGGCGGGCCGGGGGCGGGG
*c-KIT* G4	GGCGAGGAGGGGCGTGGCCGGC
*BCL-2* G4	CGGGCGCGGGAGGAAGGGGGCGGGAGC
*HRAS1* G4	TCGGGTTGCGGGCGCAGGGCACGGGCG
*HRAS2* G4	CGGGGCGGGGCGGGGGCGGGGGCG

**Table 2:** *K*_D_ of unmethylated or methylated *VEGF*, *c-KIT*, *BCL-2*, *HRAS1,* and *HRAS2* G4 DNAs for GST-SP1.

	*K*_D_ (nM)				
	*VEGF* G4	*c-KIT* G4	*BCL-2* G4	*HRAS1* G4	*HRAS2* G4
Unmethylated	14	16	12	10	10
Methylated	11	11	28	36	27

## Supplementary Materials

The following are available online, Figure S1: Binding analysis of GST-SP1 to *c-KIT*, *BCL-2*, *VEGF*, *HRAS1*, and *HRAS2* G4 DNA by SPR, Figure S2: Native-PAGE of double-stranded, unmethylated and methylated *c-KIT*, *BCL-2*, *VEGF*, *HRAS1*, and *HRAS2* G4 DNAs.
