# Supplementary material for: CpG Methylation Changes G-Quadruplex Structures Derived from Gene Promoters and Interaction with VEGF and SP1
Source: Molecules. 2018 Apr 18;23(4):944. doi: 10.3390/molecules23040944 (PMC6017926; doi:10.3390/molecules23040944)
Supplement: Supplementary file 1 [file molecules-23-00944-s001.pdf]

Supporting information

CpG Methylation Changes G-Quadruplex Structures Derived from Gene Promoters and Interaction with VEGF and SP1

Kaori Tsukakoshi, Shiori Saito, Wataru Yoshida, Shinichi Goto, and Kazunori Ikebukuro

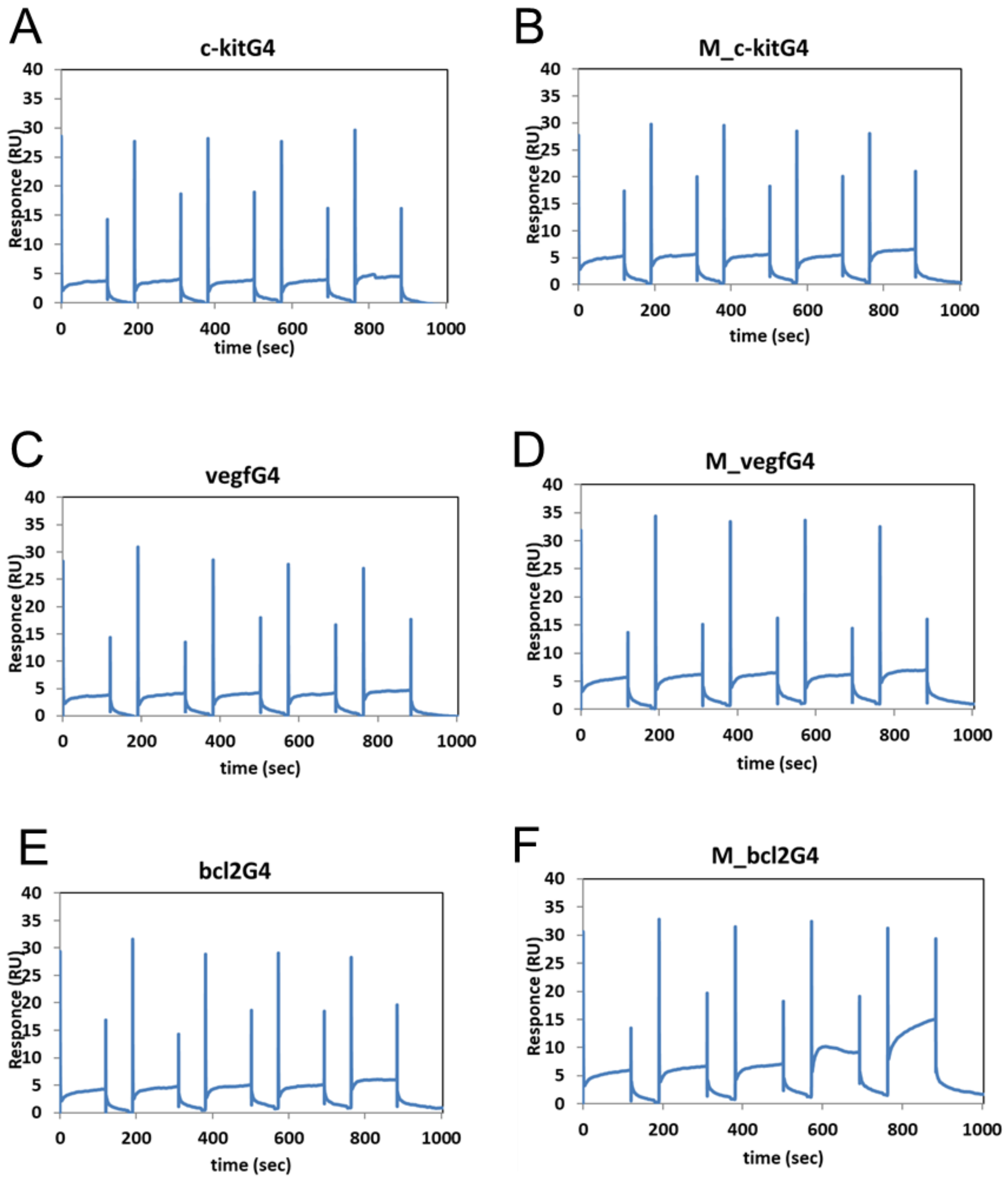
A
c-kitG4
B
M_c-kitG4
C
vegfG4
D
M_vegfG4
E
bcl2G4
F
M_bcl2G4
Responce (RU)
time (sec)
0
5
10
15
20
25
30
35
40
0
200
400
600
800
1000

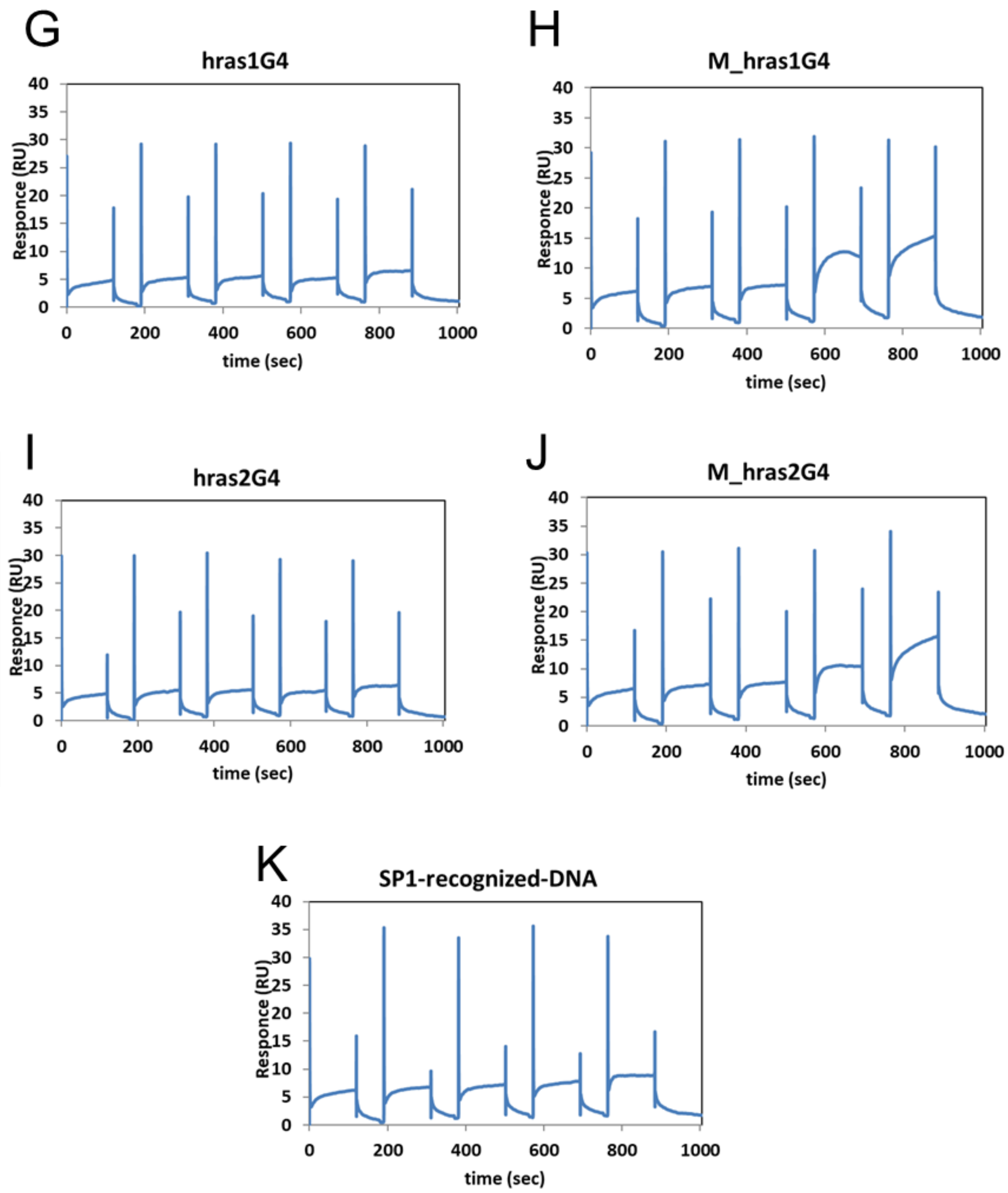

Figure S1. Binding analysis of GST-SP1 to *c-KIT*, *BCL-2*, *VEGF*, *HRAS1*, and *HRAS2* G4 DNA by SPR. Representative SPR binding signal of unmethylated (**A**) and methylated *c-KIT* G4 DNA (**B**), unmethylated (**C**) and methylated *VEGF* G4 DNA (**D**), unmethylated (**E**) and methylated *BCL-2* G4 DNA (**F**), unmethylated (**G**) and methylated *VEGF* G4 DNA (**H**), unmethylated (**I**) and methylated *VEGF* G4 DNA (**J**), or SP1-recognized dsDNA (**K**) to GST-SP1 captured by immobilized anti-GST antibodies on a CM5 chip.

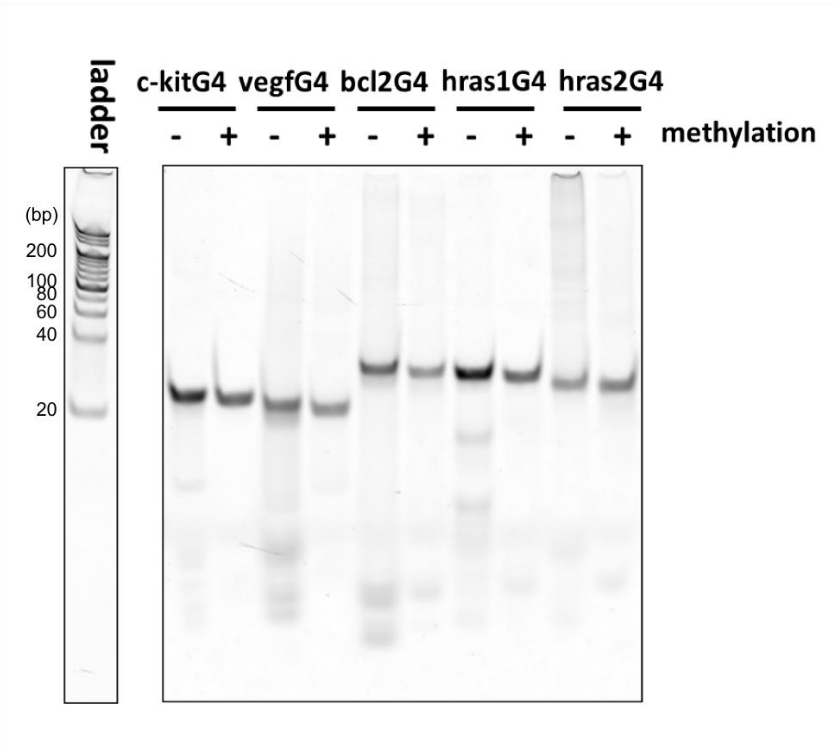

Figure S2. Native-PAGE of double-stranded, unmethylated and methylated *c-KIT*, *BCL-2*, *VEGF*, *HRAS1*, and *HRAS2* G4 DNAs. Oligonucleotides fluorescently labelled with TAMRA were detected. Ladder: DNA marker.
